# Moscatilin Inhibits Lung Cancer Cell Motility and Invasion via Suppression of Endogenous Reactive Oxygen Species

**DOI:** 10.1155/2013/765894

**Published:** 2013-05-08

**Authors:** Akkarawut Kowitdamrong, Pithi Chanvorachote, Boonchoo Sritularak, Varisa Pongrakhananon

**Affiliations:** ^1^Department of Pharmacology and Physiology, Faculty of Pharmaceutical Sciences, Chulalongkorn University, Bangkok 10330, Thailand; ^2^Cell-Based Drug and Health Product Development Research Unit, Faculty of Pharmaceutical Sciences, Chulalongkorn University, Bangkok 10330, Thailand; ^3^Department of Pharmacognosy and Pharmaceutical Botany, Faculty of Pharmaceutical Sciences, Chulalongkorn University, Bangkok 10330, Thailand

## Abstract

Lung cancer is the leading cause of death among cancer patients worldwide, and most of them have died from metastasis. Migration and invasion are prerequisite processes associated with high metastasis potential in cancers. Moscatilin, a bibenzyl derivative isolated from the Thai orchid *Dendrobium pulchellum*, has been shown to have anticancer effect against numerous cancer cell lines. However, little is known regarding the effect of moscatilin on cancer cell migration and invasion. The present study demonstrates that nontoxic concentrations of moscatilin were able to inhibit human nonsmall cell lung cancer H23 cell migration and invasion. The inhibitory effect of moscatilin was associated with an attenuation of endogenous reactive oxygen species (ROS), in which hydroxyl radical (OH^∙^) was identified as a dominant species in the suppression of filopodia formation. Western blot analysis also revealed that moscatilin downregulated activated focal adhesion kinase (phosphorylated FAK, Tyr 397) and activated ATP-dependent tyrosine kinase (phosphorylated Akt, Ser 473), whereas their parental counterparts were not detectable changed. In conclusion, our results indicate the novel molecular basis of moscalitin-inhibiting lung cancer cell motility and invasion and demonstrate a promising antimetastatic potential of such an agent for lung cancer therapy.

## 1. Introduction 

Lung cancer incidences have continued to increase worldwide [1]. More than 90% of lung cancer patients have died from metastasis because of late diagnosis after metastasis was established [2]. Treatment of metastasis lung cancer often fails due to the acquisition of chemotherapeutic resistance, and the fact that cancer metastasis is still remaining even if the tumor is removed by a surgery [3]. Recently, a number of researches have been conducted to explore potential agents for treatment of cancer metastasis. Cancer metastasis consists of multistep events facilitating the establishment of secondary tumor, in which migration and invasion play critical steps during metastasis involving the elongation of filopodia, cell contraction and gliding, and cell protrusion and reattachment to extracellular matrix [4, 5].

Aberrant generation of cellular ROS was tightly associated with several metastasis cancers such as lung and colon cancers [6, 7]. Reactive oxygen species (ROS) such as superoxide anion (O_2_^−∙^), hydrogen peroxide (H_2_O_2_), and hydroxyl radical (OH^∙^) served as important regulators of various physiological pathways during cancer metastasis including angiogenesis, cancer motility, and invasiveness [6, 8]. Scientific evidence also showed that cancer migration and invasion are obviously regulated by reactive oxygen species (ROS) [6, 8]. It was demonstrated that treatment with antioxidant such as ascorbic acid caused a reduction in cancer motility and invasion [9], and conversely the addition of exogenous ROS enhances these activities [10].

**Figure 1:**
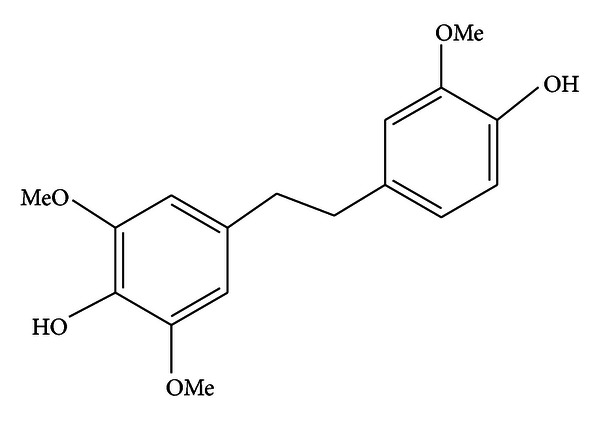
Chemical structure of moscatilin.

Moscatilin (4,4′-dihydroxy-3,3′,5′-trimethoxybibenzyl) is a bibenzyl derivative extracted from Thai orchid *Dendrobium pulchellum* (Orchidaceae), which is known as “Ueang chang nao” in Thai (Figure 1). Moscatilin was reported to have various pharmacological properties such as antiinflammatory [11], antioxidant [12], and antiplatelet aggregation [13]. Recently, it has shown anticancer activity against many kinds of cancers for example, induction of cell cycle G2-M arrest [14, 15] and cell apoptosis involving its antioxidant effect [12, 15]. In present study, we investigated that the administration of moscatilin attenuates migration and invasion in lung cancer cells. Its negative regulator is associated with the ability of compound to suppress endogenous ROS generation and consequently inhibits FAK and Akt activation-mediating cancer motility and invasiveness. Our finding reveals the novel mechanism of moscatilin on the regulation of cancer migration and invasion, which could be an advantage in development of this compound for cancer therapy.

## 2. Material and Methods

### 2.1. Cells and Reagents

 Human lung adenocarcinoma H23 cells were obtained from American Type Culture Collection (ATCC, Manassas, VA, USA), and cultured in RPMI-1640 medium containing 10% fetal bovine serum, 2 mM L-glutamine, 100 IU/mL penicillin, and 100 *μ*g/mL streptomycin (Gibco, MD, USA) in 37°C with 5% CO_2_-humidified incubator. Moscatilin was isolated from Thai orchid *Dendrobium pulchellum* as previously described [16]. Moscatilin was dissolved in DMSO and deionized water for the indicated working concentrations. The amount of DMSO in the final solution was less than 0.1% which showed no cytotoxic in H23 cells. The 3-(4,5-dimethylthiazol-2-yl)-2,5-diphenyltetrazolium bromide (MTT), Hoechst33342, propidium iodide (PI), phalloidin tetramethylrhodamine B isothiocyanate, ribonuclease A, bovine serum albumin (BSA), and dimethylsulfoxide (DMSO) were purchased from Sigma Chemical, Inc. (St. Louis, MO, USA). Annexin V Apoptosis Detection Kit was obtained from BD Biosciences (Woburn, MA, USA). Antibodies for phosphorylated Akt (S473), Akt, phosphorylated FAK (Y397), FAK, phosphorylated ERK (Thr202/Tyr204), ERK, Cdc42, *β*-actin, and peroxidase-conjugated secondary antibodies were obtained from Cell Signaling (Denvers, MA, USA).

### 2.2. Cell Viability Assay

Cells viability was determined by MTT assay as previously described [17]. Initially, cells were seeded at a density of 10^4^ cells/well onto 96-well plate overnight. After that, they were treated with various concentrations of moscatilin for 24 h. The medium was then replaced with MTT solution (5.0 mg/mL in PBS) and incubated at 37°C for 4 h. To solubilize formazan product, the medium was replaced with 100 *μ*L DMSO, and an intensity reading of the formazan product was measured at 550 nm using a microplate reader (Anthros, Durham, NC, USA). Cell viability was expressed as the percentage calculated from absorbance of MTT-treated cells relative to control cells.

### 2.3. Apoptosis Assay

Cells were seeded at a density of 10^4^ cells/well onto 96-well plate and incubated overnight for cell attachment. After the indicated treatments, cells were washed and incubated with 10 *μ*g/mL Hoechst33342 and 5 *μ*g/mL propidium iodide (PI) for 30 min. Additionally, apoptotic cells were determined using Annexin-V Staining Assay. After the indicated treatments, cells were washed and subsequently stained with 100 *μ*L of 1x binding buffer containing 5 *μ*L of Annexin V-FITC for 15 min at room temperature in the dark. Nuclei condensation and DNA fragmentation of apoptotic cells, Annexin-V-positive cells, and PI-positive necrotic cells were visualized and scored by fluorescence microscopy (Olympus IX51 with DP70) as previously described [17]. 

### 2.4. DNA Content Analysis

Cells were seeded at a density of 3 × 10^5^ cells/well onto 6-well plate and incubated overnight for cell attachment. After the indicated treatments, cells were trypsinized and fixed in 70% absolute ethanol at −20°C overnight. After washing with PBS, cells were incubated in propidium iodide solution containing 0.1% Triton-X, 1 *μ*g/mL RNase, and 1 mg/mL propidium iodide at room temperature for 30 min. DNA content was analyzed using flow cytometry (FACSort, Becton Dickinson, Rutherford, NJ, USA) as previously described [17]. 

### 2.5. Migration Determination

Migration was determined by wound healing and Boyden chamber assay as previously described [6]. For wound healing assay, cells were seeded at a density of 2 × 10^5^ cells/well onto 24-well plate. After the cell monolayer was formed, a micropipette tip was used to scratch cell attachment to generate wound space. The cells were then washed with PBS and replaced with RPMI medium containing the indicated concentration of moscatilin. The progress of cell migration into the wound was photographed by inverted microscope (Olympus IX51 with DP70) at indicated time of incubation. The average wound space was calculated from the random field of view and represented the relative cell migration. Relative cell migration was calculated by dividing the change of wound space of sample by that of the control cells in each experiment. In case of Boyden chamber assay, cells were seeded at a density of 5 × 10^4^ cells/well onto upper 24-transwell plate of the transwell filter (8-*μ*M pore) in-serum-free medium, and incubated with various concentrations of moscatilin. RPMI medium containing 10% FBS was added at lower chamber. Following the incubation, the nonmigrate cells in the upperside membrane were removed by cotton-swab wiping, and cells that migrated to the underside of the membrane were stained with 10 *μ*g/mL Hoechst33342 for 10 min, visualized and scored under a fluorescence microscope (Olympus IX51 with DP70).

### 2.6. Invasion Assay

The invasion assay was carried out using 24-transwell chambers as previously described [6], which were coated with 50 *μ*L of 0.5% matrigel on the upper surface of chamber overnight at 37°C in a humidified incubator. Following the incubation, cells were seeded at a density of 5 × 10^4^ cells/well onto upper chambers in serum-free medium containing various concentrations of moscatilin, and RPMI medium containing 10% FBS was added to the lower chamber. After the indicated time, noninvaded cells in the upperside of membrane were removed by cotton-swab wiping. Invaded cells in the underside of membrane were fixed with cold absolute methanol for 10 min and stained with 10 *μ*g/mL Hoechst33342 for 10 min. Cells were then visualized and scored under a fluorescence microscope (Olympus IX51 with DP70).

### 2.7. Cell Morphology Characterization

Cell morphology was investigated by phalloidin-rhodamine and sulforhodamine B staining assay as described in [18]. Cells were seeded at a density of 10^4^ cells/well onto 96-well plate overnight. Cells were treated with various concentrations of moscatilin for 24 h. Cells were then washed with PBS, fixed with 4% paraformaldehyde in PBS for 10 min at 37°C, permeabilized with 0.1% Triton-X100 in PBS for 4 min, and blocked with 0.2% BSA for 30 min. Cells were then incubated with either 1 : 100 phalloidin-rhodamine in PBS or 0.4% sulforhodamine B in 1% acetic acid for 15 min, rinsed 3 times with PBS, and mounted with 50% glycerol. Cell morphology was then imaged by fluorescence (Olympus IX51 with DP70). Filopodia protrusion was represented in comparison with control cells.

### 2.8. Reactive Oxygen Species Detection

Intracellular ROS were determined using specific ROS detection probe including dichlorofluorescein diacetate (DCFH_2_-DA; ROS probe), hydroxyphenyl fluorescein (HPF; specific OH^∙^ probe), amplex red (specific H_2_O_2_ probe) and dihydroethidium (DHE; specific O_2_^∙−^ probe) as previously described in [17]. After the indicated treatments, cells were incubated with either 100 *μ*M of DCFH_2_-DA, 100 *μ*M of HPF, 10 mM of amplex red, or 100 *μ*M of DHE for 30 min at 37°C, after which they were washed and immediately analyzed for fluorescence intensity using a microplate reader.

### 2.9. Western Blotting

Cells were seeded at a density of 3 × 10^5^ cells/well onto 6-well plates overnight. After specific treatment, cells were washed twice with cold PBS and incubated with lysis buffer containing 20 mM Tris-HCl (pH 7.5), 1% Triton X-100, 150 mM sodium chloride, 10% glycerol, 1 mM sodium orthovanadate, 50 mM sodium fluoride, 100 mM phenylmethylsulfonyl fluoride, and protease inhibitor cocktail (Roche Molecular Biochemicals) for 40 min on ice. Cell lysates were collected, and the protein content was determined using the BCA protein assay kit (Pierce Biotechnology, Rockford, IL, USA). Equal amounts of protein from each sample (60 *μ*g) were denatured by heating at 95°C for 5 min with Laemmli loading buffer and subsequently loaded onto a 10% SDS-polyacrylamide gel for electrophoresis. After separation, proteins were transferred onto 0.45 *μ*M nitrocellulose membranes (Bio-Rad). The transferred membranes were blocked in 5% nonfat dry milk in TBST (25 mM Tris-HCl (pH 7.5), 125 mM NaCl, 0.05% Tween 20) for 1 h, after which it was incubated with a specific primary antibody overnight at 4°C. Membranes were washed three times with TBST for 10 min and incubated with Horseradish peroxidase (HRP)-conjugated anti-rabbit or anti-mouse IgG for 2 h at room temperature. After three times of washing with TBST, the immune complexes were detected by enhancement with a chemiluminescent substrate (Supersignal West Pico; Pierce, Rockfore, IL, USA) and quantified using analyst/PC densitometry software (Bio-Rad). 

### 2.10. Statistical Analysis

All results from four or more independent experiments were presented as the mean ± standard deviation (SD). Statistical differences between the means were analyzed using one-way ANOVA with Turkey *post hoc* test at a significance level of *P* < 0.05 using SPSS version 19.0.

## 3. Results

### 3.1. Cytotoxicity of Moscatilin to H23 Cells

**Figure 2:**
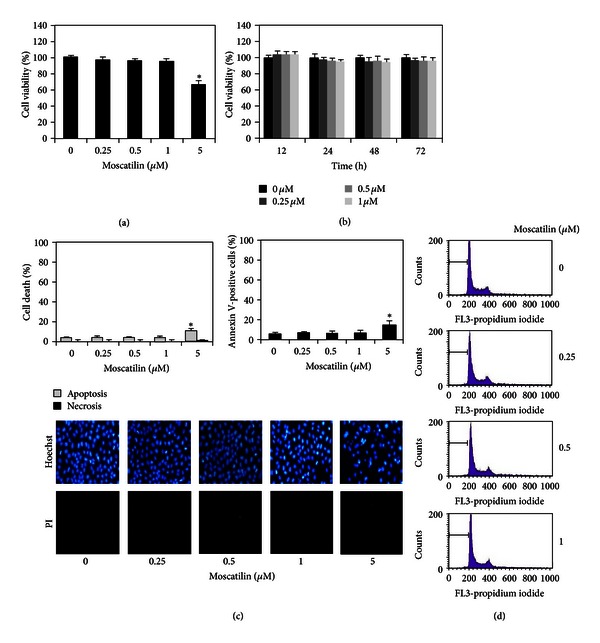
Cytotoxicity of moscatilin on human lung H23 cells. (a) Cells were treated with various concentrations of moscatilin (0–5 *μ*M) for 24 h. (b) Cells were treated with moscatilin (0-1 *μ*M) for various times (0–72 h). Cytotoxicity was determined by 3-(4,5-dimethyl-thiazol-2-yl)-2,5-diphenyl tetrazolium bromide (MTT) assay. (c) After indicated treatment for 24 h, mode of cell death was examined by Hoechst33342/PI costaining assay and Annexin-V staining assay. (d) Cellular apoptosis was determined by DNA content analysis using flow cytometry. Data represent the means ± SD (*n* = 4). *P < 0.05 versus nontreated control cells.

To investigate the inhibitory effect of moscatilin on cancer migration and invasion, prerequisite information regarding its cytotoxicity is crucial. Human lung H23 cells were treated with various concentrations of moscatilin (0–5 *μ*M) for 0–48 h, and cell viability was examined by MTT assay. Figures 2(a) and 2(b) show that a significant cytotoxic effect of moscatilin was appeared at the concentration of 5 *μ*M at 24 h, with approximately 70% of cells remaining viable, while the concentrations of such a substance less than 1 *μ*M show nontoxic effect in both dose and time studies. Hoechst33342/PI assay also confirmed that apoptosis and necrotic cell death were not found significantly in response to 0-1 *μ*M moscatilin, whereas apoptotic nuclei were appeared in the cells treated with 5 *μ*M of moscatilin, similar with the data obtained from Annexin-V staining assay (Figure 2(c)). Consistent with the above findings, DNA content analysis revealed that treatment with 0-1 *μ*M moscatilin caused no detectable change in the percentage of cells in each phase of cell cycle, compared with nontreated control (Figure 2(d)). This result suggests that lower doses of moscatilin (0-1 *μ*M) caused neither toxic nor proliferative effects on lung cancer H23 cells.

### 3.2. Effect of Moscatilin on H23 Cells Migration

**Figure 3:**
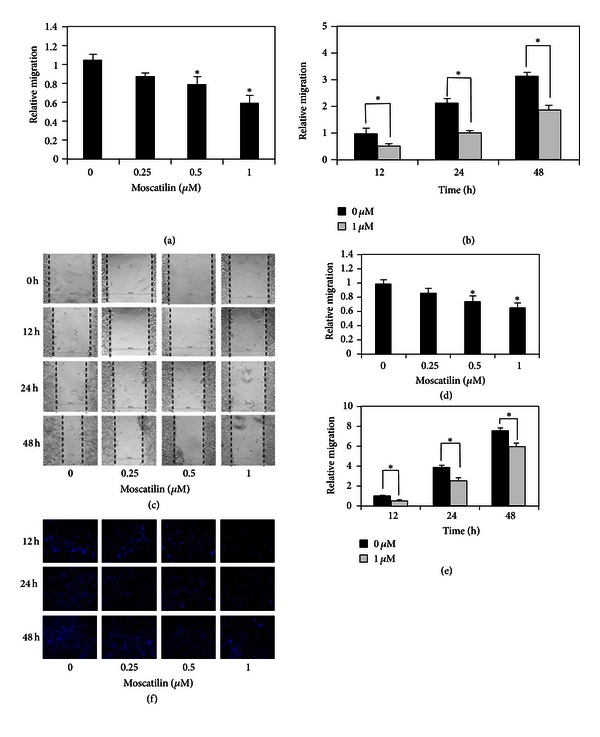
Effects of moscatilin on H23 cell migration. (a) Confluent monolayer of H23 cells was wounded using a 1 mm width tip and incubated with nontoxic dose of moscatilin (0-1 *μ*M) for 24 h. Wound space was analyzed and represented as migration level relatively to the change of those in nontreated cells. Data represent the means ± SD (*n* = 4). *P < 0.05 versus nontreated control cells. (b) Confluent monolayer of H23 cells was wounded using a 1 mm width tip and incubated with moscatilin (1 *μ*M) or without for various times (12–48 h). Wound space was analyzed and represented as migration level relatively to the change of those in nontreated cells. *P < 0.05 versus nontreated control cells. (c) After indicated treatment, migrating cells in the denuded zone were photographed. (d) H23 cell migration was examined by transwell assay for 24 h. Data were plotted as an average number of cells in each field and represented the means ± SD (*n* = 4). *P < 0.05 versus nontreated control cells. (e) Cells were treated with moscatilin (1 *μ*M) or without for various times (12–48 h) on transwell. Data were plotted as an average number of cells in each field and represented the means ± SD (*n* = 4). *P < 0.05 versus nontreated control cells. (f) Migratory cells at the basolateral side of membrane were stained with Hoechst33342 for 30 min and visualized under fluorescence microscopy.

The negative regulatory role of moscatilin on lung cancer migration was investigated by wound healing and Boyden chamber assays. Figures 3(a) and 3(c) show that treatment of the cells with nontoxic doses of moscatilin (0-1 *μ*M) inhibited migration of the cells across the wound space in a dose-dependent manner, of which approximately 0.75- and 0.55-fold of relative migration level were found in cells treated with 0.5 and 1 *μ*M, respectively, compared with nontreated control cells. In addition, moscatilin also causes antimigrative effect in a time-dependent study (Figures 3(b) and 3(c)). Boyden chamber assay supported our finding that the migrating cells on the lower side of membrane were decreased gradually in dose- and time-dependent manners (Figures 3(d), 3(e), and 3(f)). These results suggest the promising role of moscatilin in regulation of lung cancer migration. 

### 3.3. Effect of Moscatilin on H23 Cells Invasion and Filopodia Formation

**Figure 4:**
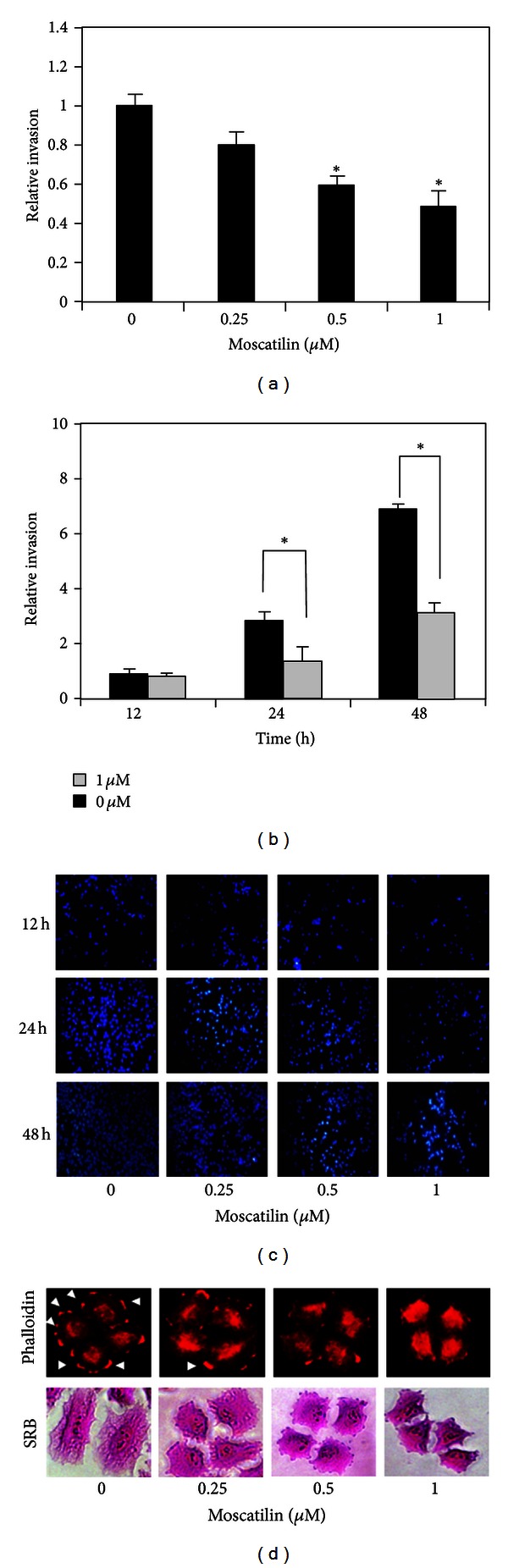
Effects of moscatilin on H23 cell invasion. (a) H23 cells were treated with various nontoxic doses of moscatilin (0-1 *μ*M) for 24 h. (b) H23 cells were treated with moscatilin (1 *μ*M) or left untreated as control for various times (12–48 h). Cell invasion was evaluated using transwell coated with matrigel as described under Section 2. Invaded cells across the membrane were stained with Hoechst33342 for 30 min. Data were plotted as an average number of cells in each field and represented the means ± SD (*n* = 4). *P < 0.05 versus nontreated control cells. (c) Invading cells were stained with Hoechst33342 and visualized under fluorescence microscopy. (d) Effect of moscatilin on filopodia formation and cell morphology. After being treated with nontoxic dose of moscatilin for 24 h, cells were stained with either phalloidin or sulforhodamine B and visualized under fluorescence microscope. Filopodia was indicated by arrow.

To further investigate the effect of moscatilin in lung cancer cell invasion, H23 cells were treated with nontoxic concentrations of moscatilin (0-1 *μ*M) for various times (0–48 h), and invaded cells were examined by transwell invasion assay. Figures 4(a) and 4(c) show that nontoxic concentrations of moscatilin retarded a number of invaded cells in a dose-dependent fashion, in which approximately 0.6- and 0.5-fold of relative invaded cells were deserved in response to 0.5 and 1 *μ*M of moscatilin, respectively. Furthermore, moscatilin was able to impede invaded cells in the time-dependent study (Figures 4(b) and 4(c)). 

Since filopodia has been shown to play an essential role in cell motility and invasion by protrusion at the edge of motile cells for attachment and gliding [5], we further clarified whether the antimigrative and antiinvasive effects of moscatilin were related to the presence of filopodia. H23 cells were treated with nontoxic concentrations of moscatilin (0-1 *μ*M) for 24 h, and filopodia of cells was identified by phalloidin-rhodamine and sulforhodamine B staining assays.  Figure 4(d) shows that, upon migration, motile cells exhibited filopodia protrusions accumulating at the cellular edge, in which these filopodia were dramatically decreased in response to moscatilin treatments. The above finding suggests that moscatilin inhibits filopodia formation, and subsequence impedes lung cancer cell migration and invasion. 

### 3.4. Moscatilin Attenuates Cell Motility through ROS-Dependent Mechanism

**Figure 5:**
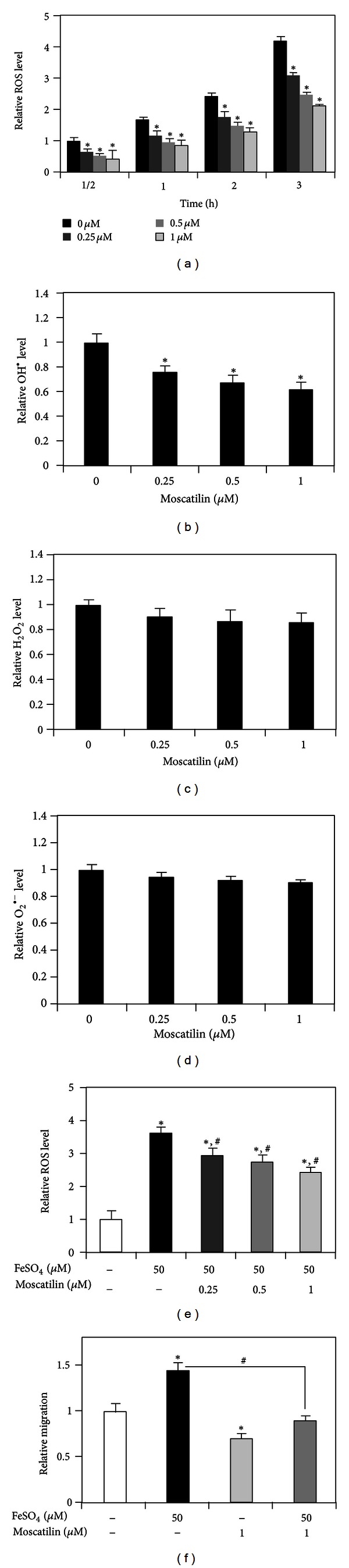
Effect of moscatilin on endogenous reactive oxygen species (ROS) generation. H23 cells were treated with various nontoxic doses of moscatilin (0-1 *μ*M) for various times (0–3 h). (a) Endogenous cellular ROS levels were determined by dichlorofluorescein diacetate (DCFH_2_-DA) probe. Values are mean ± SD (*n* = 4). *P < 0.05 versus nontreated control cells of each time point. (b) After the indicated treatment for 3 h, cells were incubated with hydroxyphenyl fluorescein (HPF) probe. Hydroxyl radical level was detected using fluorescence microplate reader. *P < 0.05 versus nontreated control cells. (c) Hydrogen peroxide level was examined using amplex red probe. *P < 0.05 versus nontreated control cells. (d) Superoxide anion level was detected by dihydroethidium (DHE) probe. *P < 0.05 versus nontreated control cells. (e) Cells were pretreated with 50 *μ*M of ferrous sulfate (FeSO_4_) for 30 min prior to moscatilin treatments (0-1 *μ*M) for 3 h. Endogenous ROS level were determined by using dichlorofluorescein diacetate (DCFH_2_-DA) probe. Values are mean ± SD (*n* = 4). *P < 0.05 versus nontreated control cells. ^#^ *P* < 0.05 versus ferrous sulfate treated cells. (f) Confluent monolayer of H23 cells was wounded using a 1 mm width tip and treated with moscatilin (1 *μ*M) in the presence or absence of 50 *μ*M of ferrous sulfate (FeSO_4_) for 24 h. Wound space was analyzed and represented as migration level relatively to the change of those in nontreated cells. *P < 0.05 versus nontreated control cells. ^#^ *P* < 0.05 versus ferrous sulfate treated cells.

It has been well documented that endogenous ROS, namely, superoxide anion, hydrogen peroxide, and hydroxyl radical are continuously produced inside the living cells [19]. Substantial studies have indicated the regulatory role of such specific ROS in cell behaviors including migration and invasion [6, 8], and most evidence indicated that these specific ROS play distinguishable roles in cell motility. In order to provide the precise mechanism of moscatilin in the regulation of cell migration, cells were treated with various concentrations of moscatilin, and cellular ROS were investigated by using DCFH_2_-DA, specific ROS detection probe. As expected, moscatilin caused a gradual decrease of endogenous ROS level in dose- and time-dependent manners (Figure 5(a)). In order to identify the specific ROS involved in our tested conditions, cells were treated with moscatilin (0-1 *μ*M) for 3 h and incubated with specific ROS detection probes: hydroxyphenyl fluorescein (HPF), amplex red and dihydroethidium (DHE). Interestingly, moscatilin shows an antioxidant effect, by which the level of OH^∙^ is substantially decreased in response to moscatilin treatment (Figure 5(b)). In addition, no change was observed regarding the level of O_2_^∙−  ^and H_2_O_2_ in comparison with nontreated control cells (Figures 5(c) and 5(d)), suggesting that endogenous OH^∙^ is a targeted species eliminated by moscatilin. To confirm the anti-OH^∙^ effect of moscatilin, cells were treated with specific OH^∙^ generator (ferrous sulfate) in the presence of moscatilin for 3 h, and ROS levels were identified using DCFH_2_-DA-specific ROS detection probes.  Figure 5(e) clearly demonstrates that an extensive increase in ROS level mediated by ferrous sulfate was in turn suppressed gradually by moscatilin in a dose-dependent fashion. This novel finding indicated that moscatilin shows a potent antioxidant against endogenous ROS, and OH^∙^ is the most affected species.

Parallel study was conducted to investigate the relevance of antioxidant effect of moscatilin on cancer migration, and cells were preincubated with OH^∙^ generator in the presence or absence of moscatilin treatment. Wound healing assay shows that ferrous sulfate treatment significantly enhanced the migration of cells, and the addition of moscatilin was able to abolish such an effect (Figure 5(f)). These findings suggest that antimigrative effect of moscatilin was associated with its ability to suppress endogenous OH^∙^. 

### 3.5. Effect of Moscatilin on the FAK Signaling in H23 Cells

**Figure 6:**
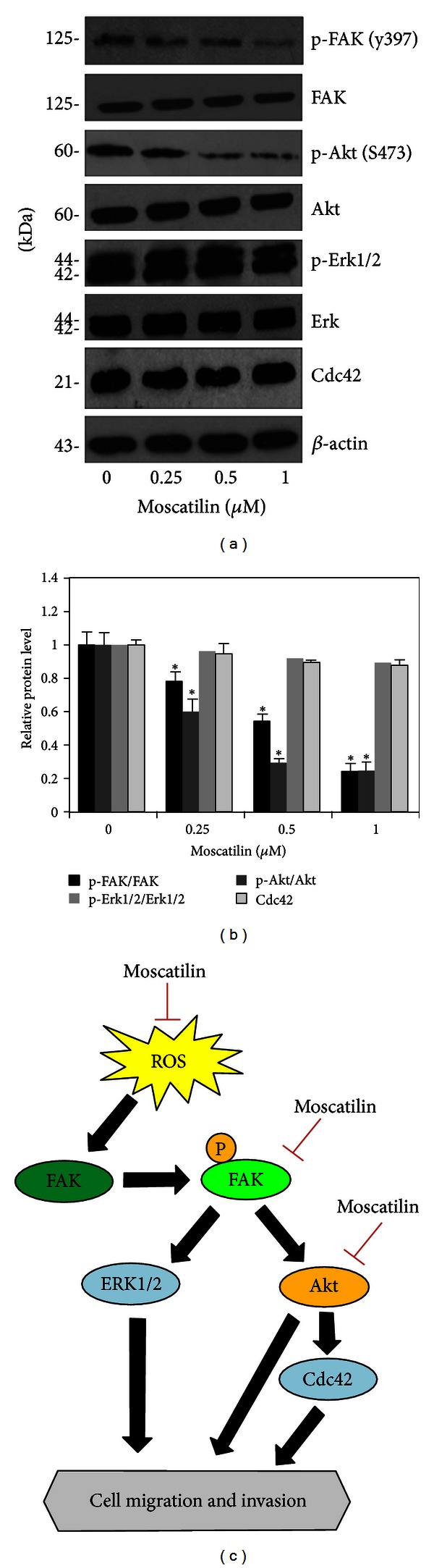
Effect of moscatilin on migratory-related proteins. (a) H23 cells was treated with various nontoxic doses of moscatilin (0-1 *μ*M) for 24 h and analyzed for protein expression by using western blot analysis as described under Section 2. Cells were collected and analyzed for phosphorylated FAK (Tyr 397), FAK proteins, phosphorylated Akt (Ser 473), Akt, phosphorylated-Erk1/2 (Thr202/Tyr204), Erk1/2, and Cdc42 proteins. Blots were reprobed with *β*-actin to confirm equal loading. (b) The immunoblot signals were quantified by densitometry and mean data from four independent experiments were presented. Values are means of samples ± SD. *P < 0.05 versus nontreated control cells. (c) A schematic diagram summarizes the inhibitory effect of moscatilin on lung cancer cell migration and invasion. Moscatilin suppresses ROS production and consequently attenuates the activation of FAK and Akt in H23 cells.

Having shown that moscatilin suppressed the migration of the cells via hydroxyl radical attenuation, we further provided the possible underlying mechanisms involving migratory regulating proteins. Focal adhesion kinase (FAK), ATP-dependent tyrosine kinase (Akt), p44/42 mitogen-activated protein kinase (ERK1/2), and cell division cycle 42 (Cdc42) were reported to implicate cell motility in several studies [5, 20, 21]; therefore, the expression and activated level of the proteins were investigated. Cells were treated with moscatilin for 24 h, and the expression levels of these proteins including activated FAK (phosphorylated FAK, Tyr 397), FAK, activated Akt (phosphorylated Akt, Ser 473), Akt, activated ERK1/2 (phosphorylated ERK1/2, Thr202/Tyr204), ERK1/2, and Cdc42 were determined by Western blotting.  Figure 6 shows that treatment with moscatilin caused a substantially downregulation of activated FAK and activated Akt as compared to nontreated control. In addition, activated ERK1/2, ERK1/2, and Cdc42 are not affected by moscatilin. These results suggest that moscatilin attenuated the activation of migrating-related proteins FAK and Akt, in accordance with the ability of such agent on lowering endogenous ROS. 

## 4. Discussion

Cancer metastasis is a complex multistep process whereby cancer cell migration and invasion are crucial in determining the capability of cancer to metastasize. Cancer migration is characterized by the movement of cancer to other places which initiates by the dynamic change of cytoskeleton including protrusion of cell membrane and actin-myosin contraction [4]. Even though the invasion of the cancer cells was shown to share certain molecular mechanisms with cell migration, invasion is more focused on the ability of cancer to disrupt basement membrane and extracellular matrix by secreting the proteolytic enzyme to destruct the meshwork of basement membrane, prior to migration through surrounding tissue [4]. Most of metastasis cancer cells exhibit these aggressive behaviors which limit the effectiveness of cancer therapy and result in high mortality rate of lung cancer patients [2]. Many studies have been conducted in the past decade to explore biological agents that have an ability to inhibit cancer metastasis. According to numerous researches, moscatilin, a major constituent of *Dendrobium pulchellum*, is one such interested in its antimutagenic activity against several cancer types [14, 15]. We also provided further evidence supporting the promising role of this natural compound for treatment of metastasis cancers. Our findings show that nontoxic doses of moscatilin were able to inhibit lung cancer cell migration and invasion (Figures 3 and 4). Our work also reported herein for the first time that such an inhibitory effect was involved with the potential of moscatilin to attenuate endogenous ROS of which OH^∙^ was identified to be an affected species. 

The role of ROS in cancer behavior has been well described including the regulation of cell motility and invasiveness [6, 10]. Recently, specific ROS, O_2_^∙−^, and H_2_O_2_ were shown to exhibit a negative regulatory effect on cell migration and invasion, whereas OH^∙^ encourages such activities [6]. Previously, moscatilin was reported to have antioxidant effect [12], and we further found that this substance reduced endogenous OH^∙^ and thus inhibited migratory action of the cells (Figure 5). Consistent with previous study, we found that the addition of ferrous sulfate promoted cancer cell motility, which can be conversed by treatment with moscatilin.

Emerging evidence showed that several signaling molecules such as focal adhesion kinase (FAK), Akt/phosphatidylinositol-3-kinase (PI3 K), and p44/42 Mitogen-activated protein kinases (ERK1/2) play enhancing roles in motility of cells [22, 23]. Recently, focal adhesion kinas (FAK) pathway has gained increasing attention as migratory-related proteins [23]. During cell motility, FAK accumulated at the site of integrin and the phosphorylated form of FAK was shown to serve as binding site for Src [20]. FAK-Src complexes enhance actin polymerization and filopodia formation through Cdc42-dependent mechanism [5, 20]. In addition, Akt and ERK signaling were implicated in cancer migration and invasion, of which the suppression of either their expressions or activity by silencing plasmid or specific inhibitor was able to attenuate these activities [21]. Accumulative studies have demonstrated that these mentioned proteins function independently from each other [24], and some evidence showed the linkage of them on cell motility [25]. FAK activation was shown to mediate Akt phosphorylation which resulted in cell movement. According to this report, the reduction of Akt activation might be a consequent event as downstream effector in response to moscatilin-attenuating FAK phosphorylation. Even ERK and Cdc42 were indicated to potentiate cells to migrate and invade [5, 23], this study demonstrated that moscatilin impeded migratory activity of H23 cells via ERK- and Cdc42-independent mechanisms. These results provide a mechanistic insight into the mechanism of moscatilin on cancer cell migration and invasion through the suppression of endogenous ROS and FAK and Akt activation.

## 5. Conclusion

 In conclusion, we reported a novel finding on moscatilin-suppressing migratory behavior of lung cancer cells and its molecular mechanism. The migrative-inhibitory effect of moscatilin was through an attenuation of endogenous OH^∙^ (Figure 6(c)). In addition, moscatilin reduced FAK and Akt activation, which, at least in part, is responsible for its antimigratory effects. Since cell motility and invasion were critical implicated in cancer metastasis, this study thus provides information and highlights potential of this natural-based compound for clinical use to overcome cancer metastasis. 

## Abbreviations

Akt: ATP-dependent tyrosine kinase
Cdc42: Cell division cycle 42
DCFH_2_-DA: Dichlorofluorescein-diacetate
DHE: Dihydroethidium
ERK1/2: Extracellular signal-regulated kinase
FAK: Focal-adhesion kinase
HPF: Hydroxyphenyl fluorescein
H_2_O_2_: Hydrogen peroxide
MTT: 3-(4,5-Dimethylthiazol-2-yl)-2,5-diphenyltetrazolium bromide
OH^∙^: Hydroxyl radical
O_2_^∙−^: Superoxide
p-Akt: Phosphorylated Akt
PBS: Phosphate-buffered saline
p-ERK1/2: Phosphorylated-ERK1/2
p-FAK: Phosphorylated-FAK
PI: Propidium iodide
ROS: Reactive oxygen species
TBST: Tris-buffered saline with 0.1% Tween.
